# Nicotinamide phosphoribosyltransferase postpones rat bone marrow mesenchymal stem cell senescence by mediating NAD^+^–Sirt1 signaling

**DOI:** 10.18632/aging.101993

**Published:** 2019-06-07

**Authors:** Chenchen Pi, Yue Yang, Yanan Sun, Huan Wang, Hui Sun, Mao Ma, Lin Lin, Yingai Shi, Yan Li, Yulin Li, Xu He

**Affiliations:** 1 The Key Laboratory of Pathobiology, Ministry of Education, College of Basic Medical Sciences, Jilin University, Changchun 130021, China; 2 The First Hospital, Jilin University, Changchun, Jilin 130021, China; 3 Sichuan Academy of Medical Sciences and Sichuan Provincial People's Hospital, Chengdu, Sichuan 610072, China; 4 Department of Pathology, Zhongda Hospital, School of Medicine, Southeast University, Nanjing, China; 5 Division of Orthopedics and Biotechnology, Department for Clinical Intervention and Technology (CLINTEC), Karolinska Institutet, Stockholm, Sweden

**Keywords:** Nampt, mesenchymal stem cells (MSC), senescence, NAD ^+^, Sirt1, regenerative medicine

## Abstract

*In vitro* replicative senescence affects MSC characteristics and functionality, thus severely restricting their application in regenerative medicine and MSC-based therapies. Previously, we found that MSC natural senescence is accompanied by altered intracellular nicotinamide adenine dinucleotide (NAD^+^) metabolism, in which Nampt plays a key role. However, whether Nampt influences MSC replicative senescence is still unclear. Our study showed that Nampt expression is down-regulated during MSC replicative senescence. Nampt depletion via a specific Nampt inhibitor FK866 or Nampt knockdown in early passage MSCs led to enhanced senescence as indicated by senescence-like morphology, reduced proliferation, and adipogenic and osteogenic differentiation, and increased senescence-associated-β-galactosidase activity and the expression of the senescence-associated factor p16^INK4a^. Conversely, Nampt overexpression ameliorated senescence-associated phenotypic features in late passage MSCs. Further, Nampt inhibition resulted in reduced intracellular NAD^+^ content, NAD^+^/NADH ratio, and Sirt1 activity, whereas overexpression had the opposite effects. Exogenous intermediates involved in NAD^+^ biosynthesis not only rescued replicative senescent MSCs but also alleviated FK866-induced MSC senescence. Thus, Nampt suppresses MSC senescence via mediating NAD^+^-Sirt1 signaling. This study provides novel mechanistic insights into MSC replicative senescence and a promising strategy for the severe shortage of cells for MSC-based therapies.

## Introduction

Adult stem cells (SCs), which reside in various tissues and organs, are critical for homeostasis maintenance and tissue regeneration. Nevertheless, SCs are prone to entering a senescent state during aging [1]. Accordingly, the aging of SCs is crucially implicated in individual aging [2,3]. Individual aging, aging of tissues and organs, and the occurrence of age-related diseases, such as diabetes, atherosclerosis, and Alzheimer’s disease have been attributed to SC senescence [4–8]. Hence, methods to rejuvenate senescent SCs need to be developed.

Because of the distinct advantages of enhanced self-renewal, multi-lineage differentiation and the avoidance of ethical controversy, adult bone marrow mesenchymal SCs (MSCs) are ideal seeding cells for tissue engineering and regenerative medicine. Primary cells cultivated *in vitro* have a finite proliferation capability termed Hayflick limit before they terminally differentiate and cease to proliferate, but the cells are still viable and metabolically active [9,10]. This condition is defined as replicative senescence, a telomere-based mechanism, as opposed to stress-induced senescence, which is achieved by exposing cells to a range of sublethal harmful agents such as toxins, radiation, chemotherapy, and oxidants [11–14]. Regardless of the donor’s age, MSCs cultured *in vitro* will inevitably senesce with an increasing number of passages, and this has adverse effects on the amplification and functionality of cells. Ultimately, this contributes to the paucity of seeding cells for SC-based therapies and severely restricts their application in basic scientific research, tissue repair, autotransplantation, and the treatment of clinical diseases. However, the molecular mechanisms underlying MSC replicative senescence are still not fully elucidated.

The mammalian aging theory “NAD^+^ world” proposed in 2009 suggests that nicotinamide phosphoribosyltransferase (Nampt), known as the rate-limiting enzyme in the NAD^+^ salvage pathway, directly determines NAD^+^ levels and silent information regulator 2 ortholog (Sirt1) activity, which play crucial roles in cell metabolism, cellular senescence, cell cycle maintenance, and individual aging [15,16]. Nampt over-expression in mouse embryonic fibroblast (MEF) cells can slow down cellular senescence by upregulating Sirt1 activity. The consequent progressive decline in Nampt can promote cellular senescence through the NAD^+^-Sirt1 pathway in retinal pigment epithelium (RPE) [17,18]. Therefore, Nampt regulation has been recognized as an essential approach to slowing down aging. Although so far research on Nampt-mediated cellular senescence has focused mainly on somatic cells [19–21], scientific investigation on whether Nampt influences SC senescence has been scarce and the functional effects of Nampt on MSC senescence await specific clarification.

In our previous study, we demonstrated that senescence is associated with a passage-dependent reduction in Nampt expression, which occurred when MSCs were serially expanded *in vitro.* Consistently, in a rat model of aging, Nampt expression was significantly lower in MSCs obtained from aged rats than in those acquired from young rats [22]. These findings suggested that Nampt likely plays a pivotal role in the regulation of MSC senescence. In addition, we previously discovered that the Nampt–NAD^+^–Sirt1 axis might participate in MSC osteoblast cell fate determination and that Nampt might serve as a marker of intracellular NAD^+^ metabolism [23]. Therefore, we hypothesized that the regulatory effects of Nampt on MSC replicative senescence might be related to NAD^+^ metabolism by mediating NAD^+^–Sirt1 signaling pathway. In the current study, we investigated the functional effects of Nampt on MSC senescence through pharmacological inhibition and gene manipulation. In addition, the possible regulatory mechanism of Nampt was further explored by measuring intracellular NAD^+^ content, NAD^+^/ NADH ratio, and Sirt1 activity.

## RESULTS

### Senescence-associated alterations in MSCs at late passage are associated with reduced Nampt expression and attenuated NAD^+^-Sirt1 signaling

**Figure 1:**
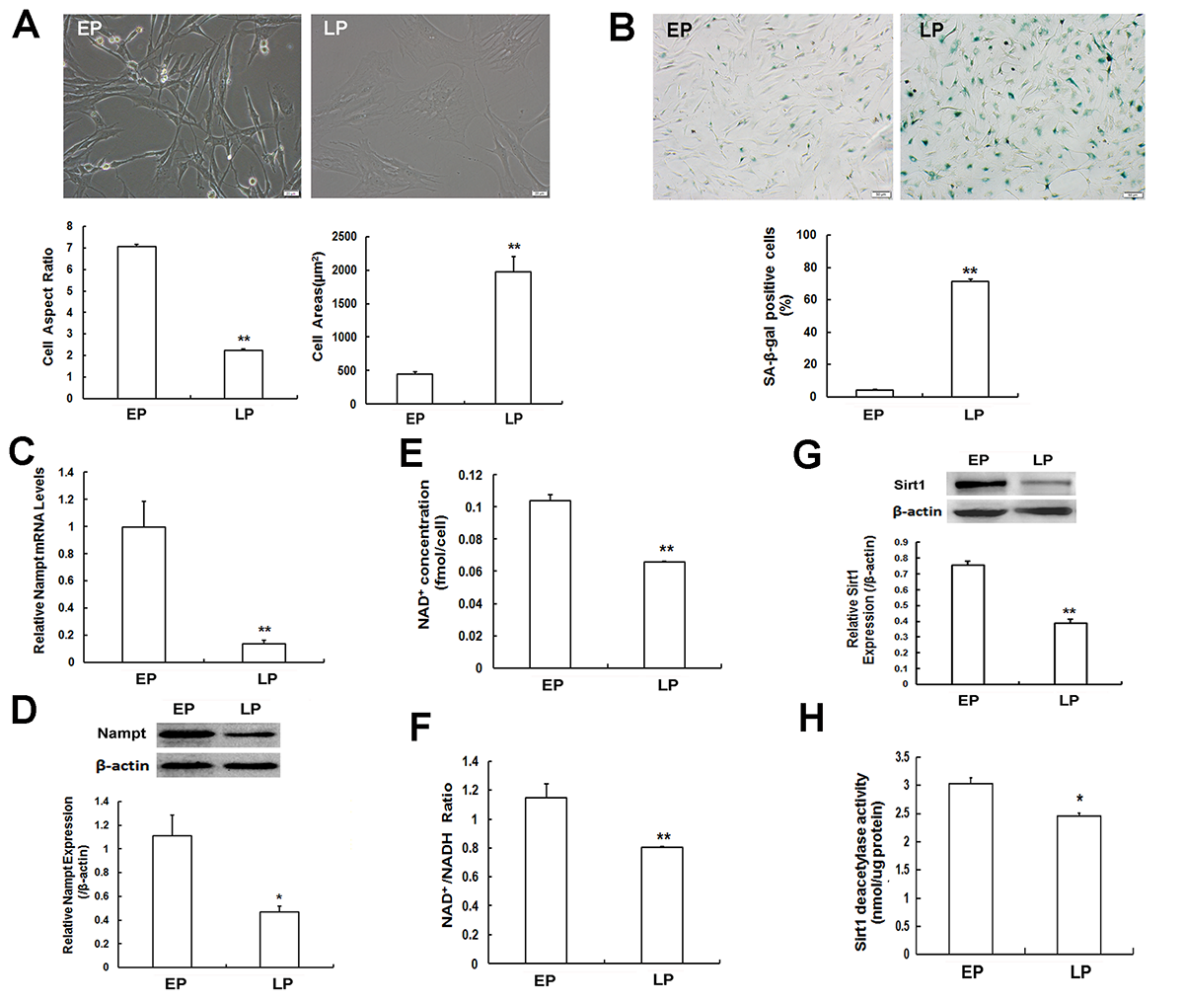
**Senescence-associated variations in mesenchymal stem cells (MSCs) and measurement of Nampt expression and NAD^+^-Sirt1 signaling. (A)** Morphological characteristics of young MSCs at early passage (EP, P3) and replicative senescent MSCs at late passages (LP, P10) (Scale bar = 20 μm) and analysis of cell aspect ratio and cell surface area. (**B)** SA-β-gal staining (scale bar = 50 μm) and quantitative analysis of SA-β-gal-positive cells. **(C)** Nampt mRNA expression determined by RT-qPCR. **(D)** Nampt protein expression determined by western blotting. **(E, F)** Detection of intracellular NAD^+^ concentration **(E)** and NAD^+^/ NADH ratio **(F)**. **(G, H)** Sirt1 protein expression evaluated by western blotting **(G)** and detection of Sirt1 deacetylase activity **(H)**; n = 3 independent experiments. *P < 0.05, **P < 0.01.

In the present study, we generated senescent MSCs via serial expansion *in vitro*. Rat MSCs at early passage (EP, P3) and late passage (LP, P10) displayed obvious morphological differences. MSCs at EP grew well and exhibited the elongated spindle shapes, whereas MSCs at LP displayed senescence-like morphology with irregular shapes, enlarged and flattened cell bodies, and noticeable particles in the cytoplasm. Quantitative analysis of cell morphology revealed that the cell aspect ratio gradually decreased, whereas the cell surface area progressively increased with passages (Fig. 1A). Senescence-associated-β-galactosidase (SA-β-gal) staining is the most widely used method for assessing senescence [24–26]. As shown in Fig. 1B, a small number of blue-stained MSCs were observed at EP, whereas this population was augmented at LP. Quantitative analysis indicated that the ratio of SA-β-gal-positive cells in LP MSCs was significantly elevated when compared to that in EP MSCs. These results demonstrated that LP MSCs displayed the senescent changes unlike EP MSCs, and replicative senescence occurred with increasing passages.

To determine the potential role of Nampt in MSC replicative senescence, we detected its expression by Real-time quantitative polymerase chain reaction (RT-qPCR) and western blotting. At both mRNA (Fig. 1C) and protein (Fig. 1D) levels, Nampt expression was significantly decreased in LP MSCs compared to that in EP cells. Based on the NAD^+^ world theory, declined Nampt expression is linked to the age-related down-regulation of intracellular NAD^+^ levels and Sirt1 deacetylase activity [27]. Accordingly, we next examined NAD^+^-Sirt1 signaling pathway in both cells. The results indicated that intracellular NAD^+^ content (Fig. 1E), NAD^+^/NADH ratio (Fig. 1F), Sirt1 protein expression (Fig. 1G) and Sirt1 deacetylase activity (Fig. 1H) in LP MSCs were substantially lower than those in EP MSCs. Taken together, our data showed that Nampt might be involved in the regulation of MSC replicative senescence via NAD^+^-Sirt1 signaling.

### Nampt depletion induces MSC senescence at early passage

**Figure 2:**
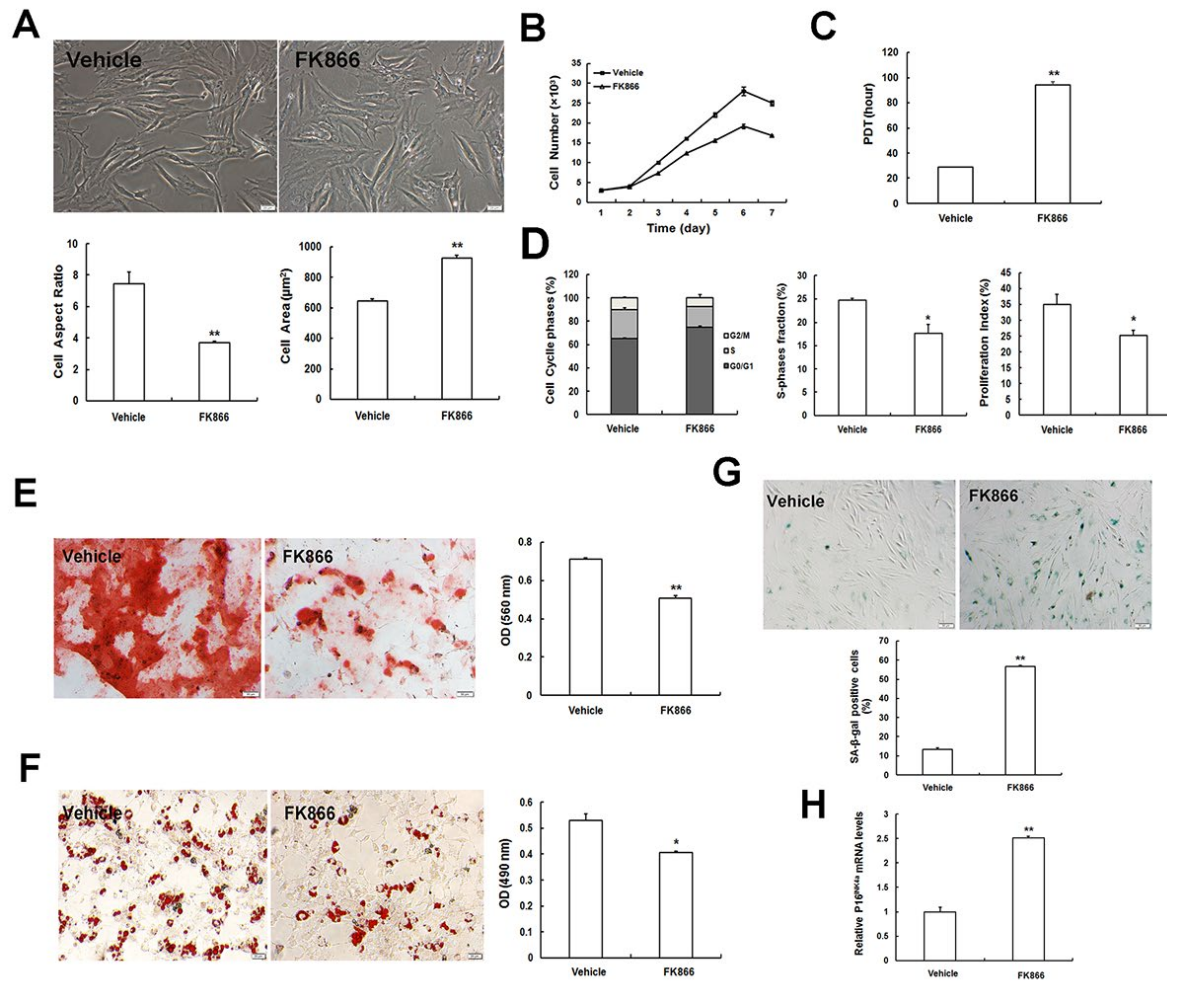
**The specific Nampt inhibitor FK866 induces cellular senescence. (A)** Morphological appearance (scale bar = 20 μm) and quantification in young EP MSCs (P3) when treated with FK866. **(B)** Cell growth curves. **(C)** Population doubling time (PDT). **(D)** Evaluation of cell cycle by flow cytometry, and analysis of S-phase fraction (SPF) and proliferation index (PI). **(E)** Osteogenic differentiation of MSCs determined by Alizarin Red S staining (scale bar = 50 μm). **(F)** Adipogenic differentiation of MSCs determined by Oil red O staining (Scale bar = 20 μm). **(G)** SA-β-gal staining of MSCs (scale bar = 50 μm) and quantification. **(H)** mRNA expression of the senescence marker p16^INK4a^; n = 3 independent experiments. *P < 0.05, **P < 0.01.

To investigate whether Nampt directly affects MSC senescence, we first evaluated the effect of a specific Nampt inhibitor, FK866, on MSC senescence. We found that at concentrations higher than 12.5 nM, FK866 exerted a cytotoxic effect on young EP MSCs (P3) (data not shown). Therefore, P3 MSCs were treated with 10 nM FK866 in subsequent experiments. In the presence of FK866, P3 MSCs presented senescent morphology with enlarged and flattened cell bodies, blurred cell borders, and clearly visible particles in the cytoplasm (Fig. 2A). The cell aspect ratio was markedly reduced, and the cell surface area was enlarged (Fig. 2A). In addition, cell proliferation declined (Fig. 2B) and the population doubling time (PDT) was significantly prolonged after FK866 treatment (Fig. 2C). Cell cycle analysis revealed that FK866 induced cell cycle arrest in G1 phase, and the S-phase fraction (SPF) and proliferative index (PI) were lower in the FK866 group than those in the vehicle group (Fig. 2D). Multilineage differentiation is one of the most important hallmarks of SCs; therefore, we next tested whether FK866 could influence MSC osteogenic and adipogenic differentiation. After the treatment of FK866, matrix mineralization was reduced as indicated by Alizarin red S staining (Fig. 2E), and lipid droplet formation was diminished as indicated by oil red O staining (Fig. 2F). Quantitative analysis indicated that MSC osteogenesis and adipogenesis were significantly attenuated by the addition of FK866. Furthermore, both SA-β-gal activity (Fig. 2G) and p16^INK4A^ expression (Fig. 2H) were evidently elevated in the FK866 group compared to those in the vehicle group.

**Figure 3:**
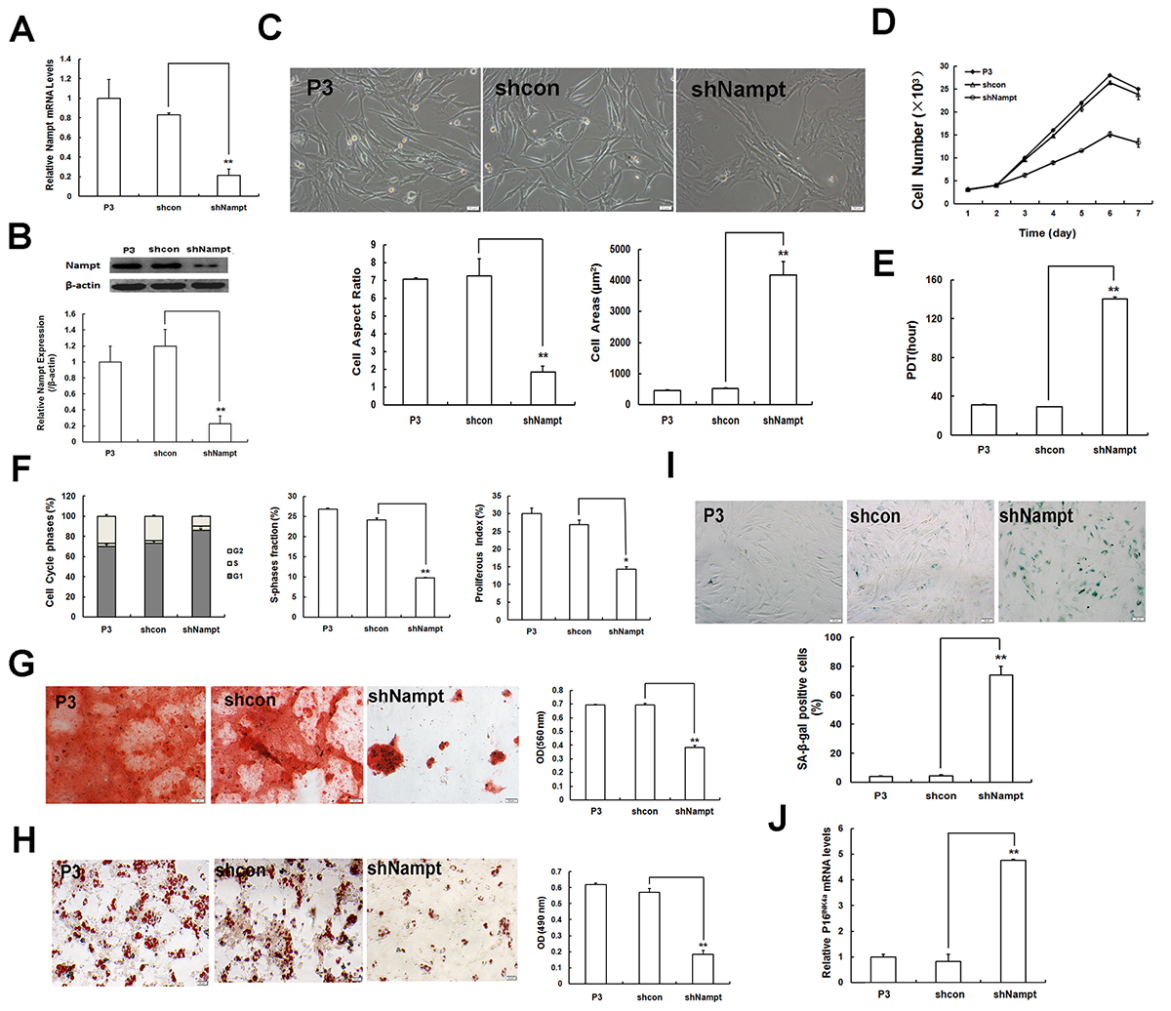
**Nampt gene silencing exacerbates MSC senescence. (A)** Gene expression of Nampt in young EP MSCs (P3) after Nampt knockdown as demonstrated by RT-qPCR. **(B)** Protein levels of Nampt as detected by western blotting. **(C)** Senescent morphology (scale bar = 20 μm) and quantification. **(D)** Logarithmic proliferation in Nampt-deficient MSCs. **(E)** Cell population doubling time (PDT). **(F)** Detection of cell cycle and analysis of both S-phase fraction (SPF) and proliferation index (PI). **(G)** Osteogenic differentiation of MSCs (scale bar = 50 μm). **(H)** Adipogenic differentiation of MSCs (scale bar = 20 μm). **(I)** SA-β-gal staining (scale bar = 50 μm) and quantification. **(J)** Gene expression of senescence-related factor p16^INK4a^; n = 3 independent experiments. *P < 0.05, **P < 0.01.

To confirm the effect of Nampt depletion on MSC senescence, we next generated Nampt-deficient MSCs via viral transduction of Nampt shRNA (shNampt) into P3 MSCS. The transduction efficacy was evaluated by RT-qPCR and western blotting. We discovered that Nampt expression at both mRNA (Fig. 3A) and protein (Fig. 3B) levels showed a significant reduction compared to that in cells transduced with non-targeting control shRNA (shcon) lentiviral particles. Accordant with the effect of FK866, Nampt deficiency in P3 MSCs induced senescence-like morphological alterations, including a reduced cell aspect ratio and enlarged cell surface area (Fig. 3C). Moreover, cell proliferation slowed down (Fig. 3D) and the PDT was increased by approximately 5-fold (Fig. 3E). The majority of cells were arrested in G1 phase, and both SPF and PI were notably repressed (Fig. 3F). In addition, both osteogenic (Fig. 3G) and adipogenic (Fig. 3H) differentiation potentials were significantly diminished after knockdown of Nampt, as indicated by decreased bone matrix mineralization and reduced intracellular lipid droplets. Furthermore, SA-β-gal activity in Nampt-depleted MSCs was markedly enhanced (Fig. 3I), and p16^INK4A^ expression was elevated (Fig. 3J). Taken together, the above data suggested that Nampt depletion in young MSCs via either pharmacological inhibition or gene silencing can promote or accelerate MSC senescence.

### Nampt repletion alleviates MSC senescence at late passage

**Figure 4:**
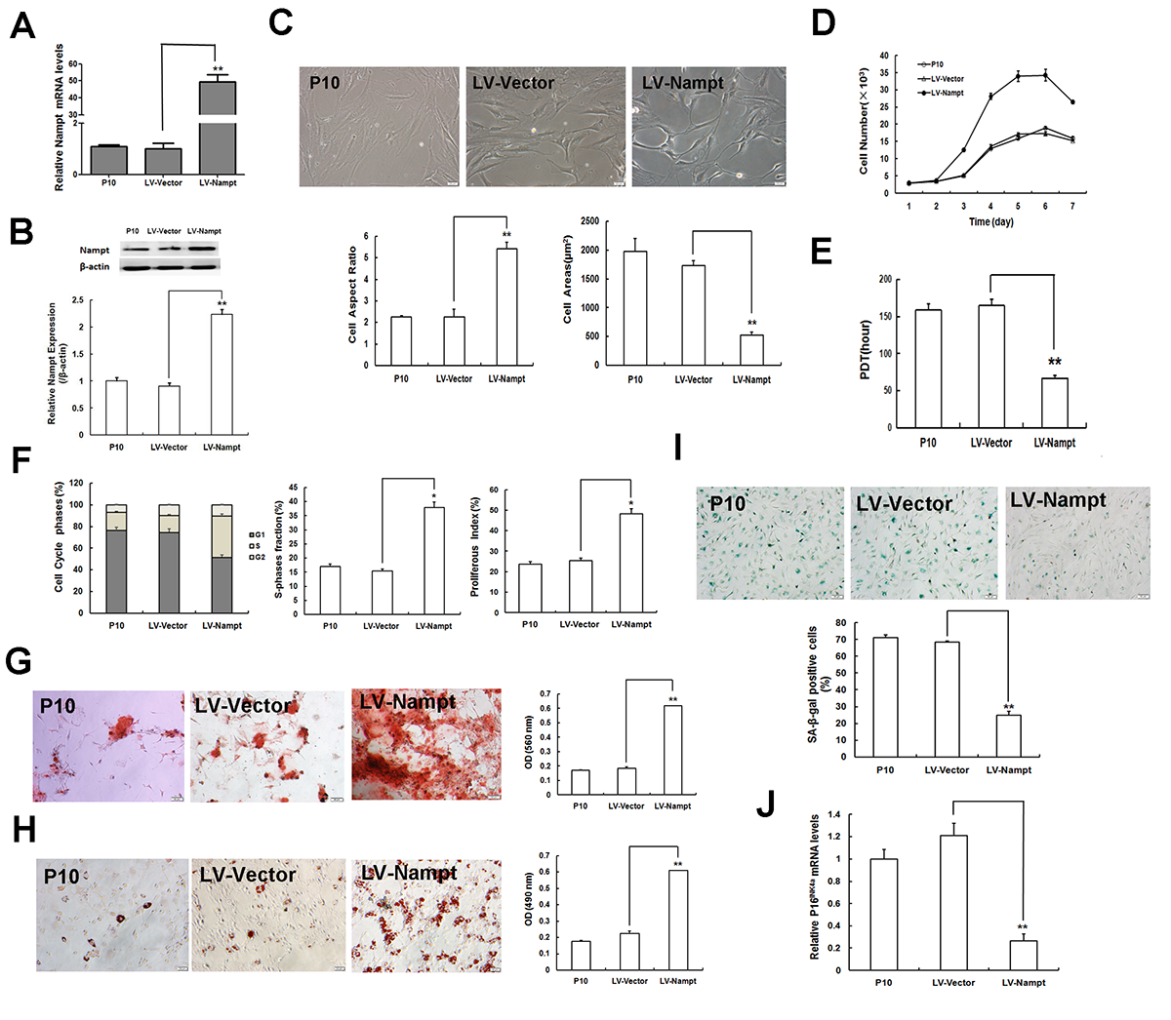
**Nampt overexpression alleviates MSC senescence. (A)** Nampt mRNA expression determined by RT-qPCR in senescent LP MSCs (P10) after Nampt overexpression. **(B)** Nampt protein expression examined by western blotting. **(C)** Cellular morphology (scale bar = 20 μm) and quantification. **(D)** Logarithmic proliferation in Nampt-overexpressing MSCs. **(E)** Cell population doubling time (PDT). **(F)** Detection and analysis of cell cycle. **(G)** Observation of osteogenesis (scale bar = 50 μm) and quantification. **(H)** Observation of adipogenesis (scale bar = 20 μm) and quantification. **(I)** SA-β-gal staining (scale bar = 50 μm) and quantification. **(J)** Gene expression of the senescence-related factor p16^INK4a^; n = 3 independent experiments. *P < 0.05, **P < 0.01.

Considering that Nampt was expressed at low levels in senescent LP MSCs (P10), we examined whether MSC senescence could be attenuated by enforcing Nampt expression. For this purpose, P10 MSCs were transduced with lentivirus expressing Nampt (LV-Nampt) and the lentiviral vector (LV-Vector). As indicated by the results of RT-qPCR and western blotting, Nampt was successfully over-expressed at both mRNA (Fig. 4A) and protein (Fig. 4B) levels. Moreover, cellular morphology was notably altered by Nampt overexpression (Fig. 4C). Senescent cells with enlarged and flattened cell bodies became substantially smaller and slenderer, and the cell borders became distinct. The cell aspect ratio markedly increased, whereas the cell surface area prominently decreased. On the other hand, Nampt-replenished MSCs grew faster (Fig. 4D), and the PDT was shortened (Fig. 4E). The percentage of cells arrested in G1 phase was also markedly reduced by Nampt repletion, and both SPF and PI were up-regulated (Fig. 4F). Of note, matrix mineralization was largely enhanced (Fig. 4G) and lipid droplet formation was improved (Fig. 4H) following Nampt overexpression. Quantitative analysis indicated that osteogenesis and adipogenesis in senescent LP MSCs were significantly augmented after increasing Nampt expression. Moreover, both SA-β-gal activity (Fig. 4I) and p16^INK4A^ expression (Fig. 4J) in Nampt over-expressed MSCs were substantially inhibited compared to those in cells transduced with the vector. These data suggested that Nampt overexpression in senescent MSCs results in the alleviation of cellular senescence, indicating that Nampt replenishment can suppress or delay MSC senescence.

### Nampt-regulated MSC replicative senescence is mediated by NAD^+^–Sirt1 signaling

**Figure 5:**
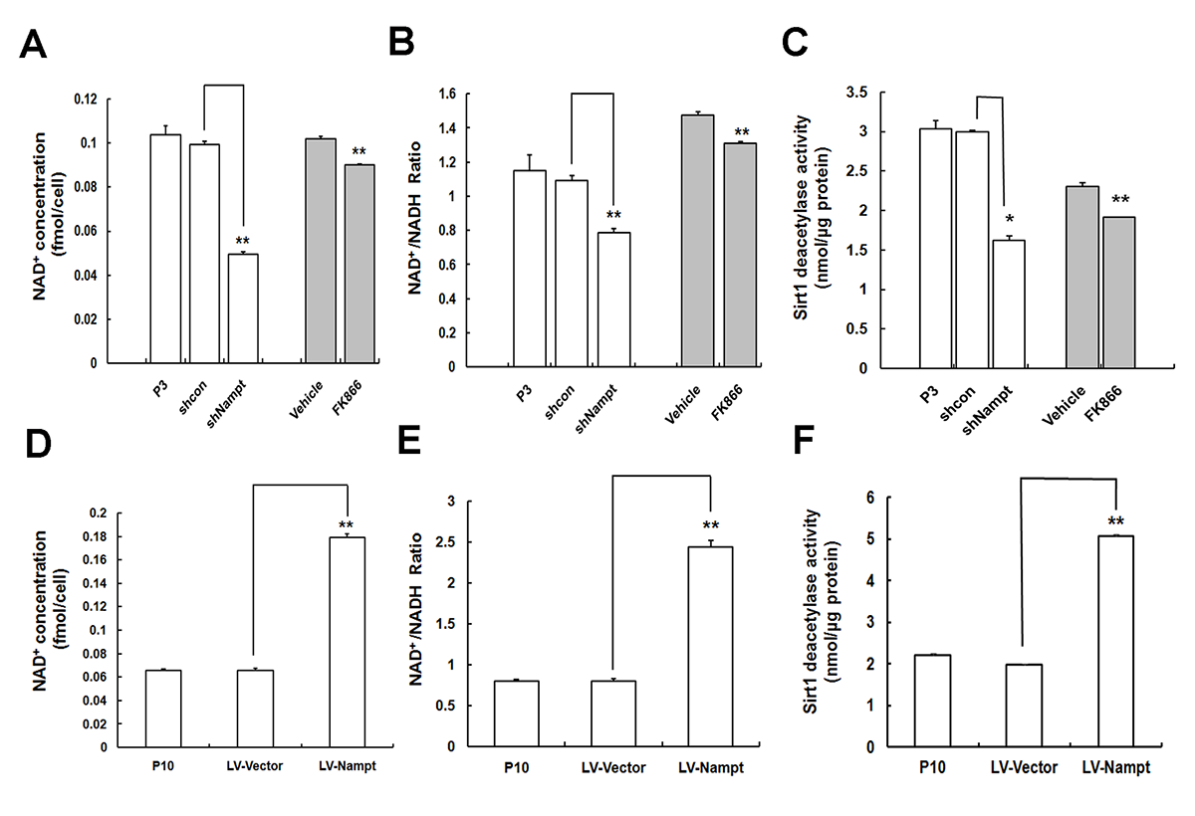
**Nampt regulation on MSC senescence is linked to the NAD^+^–Sirt1 signaling pathway. (A, B, C)** Intracellular NAD^+^ content **(A)** NAD^+^/ NADH ratio **(B)** and Sirt1 deacetylase activity **(C)** were measured when Nampt was suppressed by either FK866 treatment or gene silencing. **(D, E, F)** Effect of Nampt overexpression on intracellular NAD^+^ content **(D)** and NAD^+^/ NADH ratio **(E)**, as well as Sirt1 deacetylase activity **(F)**; n = 3 independent experiments. *P < 0.05, **P < 0.01.

It has been elucidated that Nampt can decrease age-related MSC senescence via the NAD^+^-Sirt1 axis [22]. In the present study, our results above have already proved that Nampt plays a regulatory role in MSC replicative senescence. To further find out the underlying mechanisms in this process, we attempted to investigate whether Nampt regulation on MSC replicative senescence was also mediated by NAD^+^–Sirt1 signaling pathway. To this end, we next examined the alterations in NAD^+^-Sirt1 signaling resulted from Nampt depletion and repletion in MSCs. As shown in Fig. 5, although there was no significant change in Sirt1 protein expression after FK866 treatment or Nampt overexpression in MSCs (Supplementary Figure 1A and B), Nampt deficiency via either knockdown or FK866 led to significantly lower intracellular NAD^+^ content (Fig. 5A), NAD^+^/NADH ratio (Fig. 5B) and lower Sirt1 activity (Fig. 5C) than those in the control group (shcon or Vehicle). However, Nampt overexpression conversely resulted in markedly elevated intracellular NAD^+^ content (Fig. 5D) and NAD^+^/NADH ratio (Fig. 5E), as well as increased Sirt1 deacetylase activity (Fig. 5F). These findings revealed that the mechanisms underlying Nampt-regulated MSC replicative senescence are linked to the NAD^+^–Sirt1 signaling pathway.

### NAD intermediates treatment attenuates MSC senescence via enhancing NAD^+^ biosynthesis and Sirt1 activity

**Figure 6:**
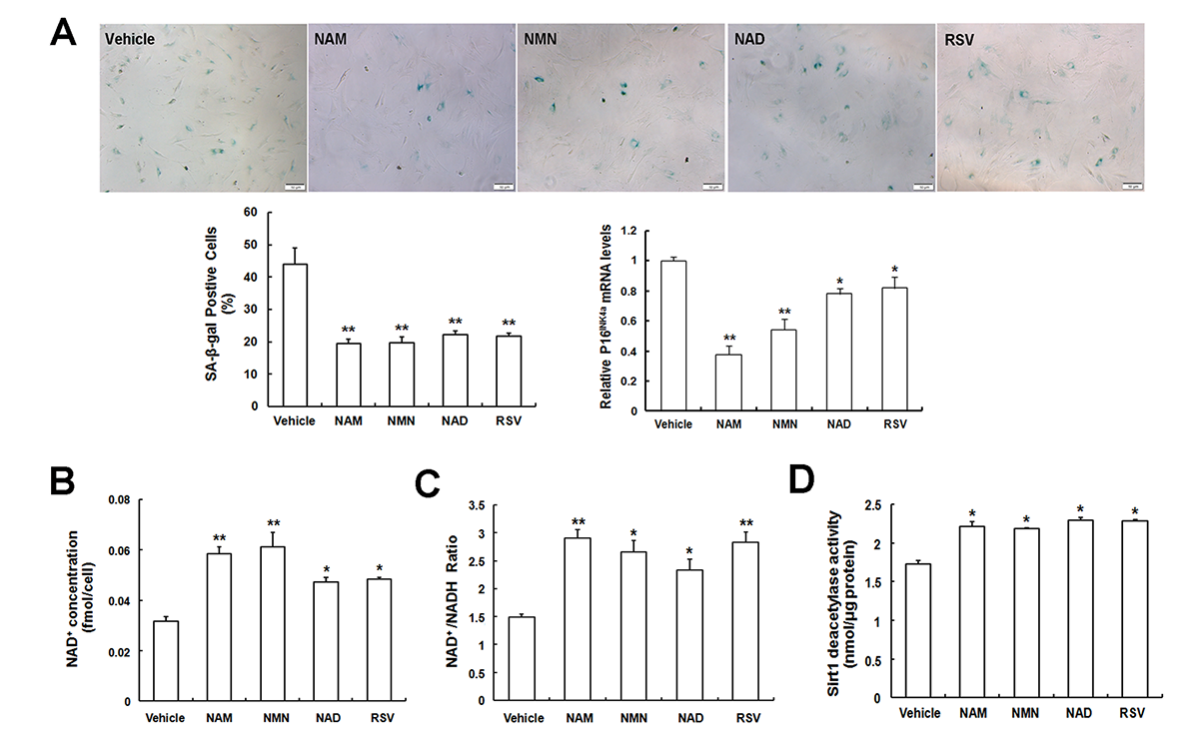
**Exogenous intermediates participated in NAD^+^ biosynthesis ameliorates MSC replicative senescence. (A)** SA-β-gal staining and p16^INK4A^ mRNA expression of senescent LP MSCs in the presence of Vehicle (DMSO treated), 100 μM NAM, 100 μM NMN, 100 μM NAD, and 5 μM resveratrol (RSV) (scale bar = 50 μm) and quantification. **(B, C, D)** Effect of NAD intermediates treatment on NAD^+^ content **(B)** and NAD^+^/ NADH ratio **(C)**, as well as Sirt1 deacetylase activity **(D)**; n = 3 independent experiments. *P < 0.05, **P < 0.01.

To further verify that Nampt-regulated MSC senescence was associated with NAD^+^–Sirt1 signaling, we assessed the effects of different exogenous intermediates in the NAD^+^ salvaging pathway on MSC senescence. First, senescent LP MSCs were treated with different concentrations of NAD intermediates or Vehicle. As shown in Fig. 6A, the ratios of SA-β-gal-positive cells as well as p16^INK4A^ mRNA expression were markedly decreased after treatment with 100 μM nicotinamide (NAM), 100 μM nicotinamide mononucleotide (NMN), 100 μM NAD, or 5 μM resveratrol (RSV). In addition, intracellular NAD^+^ content (Fig. 6B), NAD^+^/NADH ratio (Fig. 6C), and Sirt1 activity (Fig. 6D) in senescent LP MSCs were significantly elevated in response to the addition of NAD intermediates.

**Figure 7:**
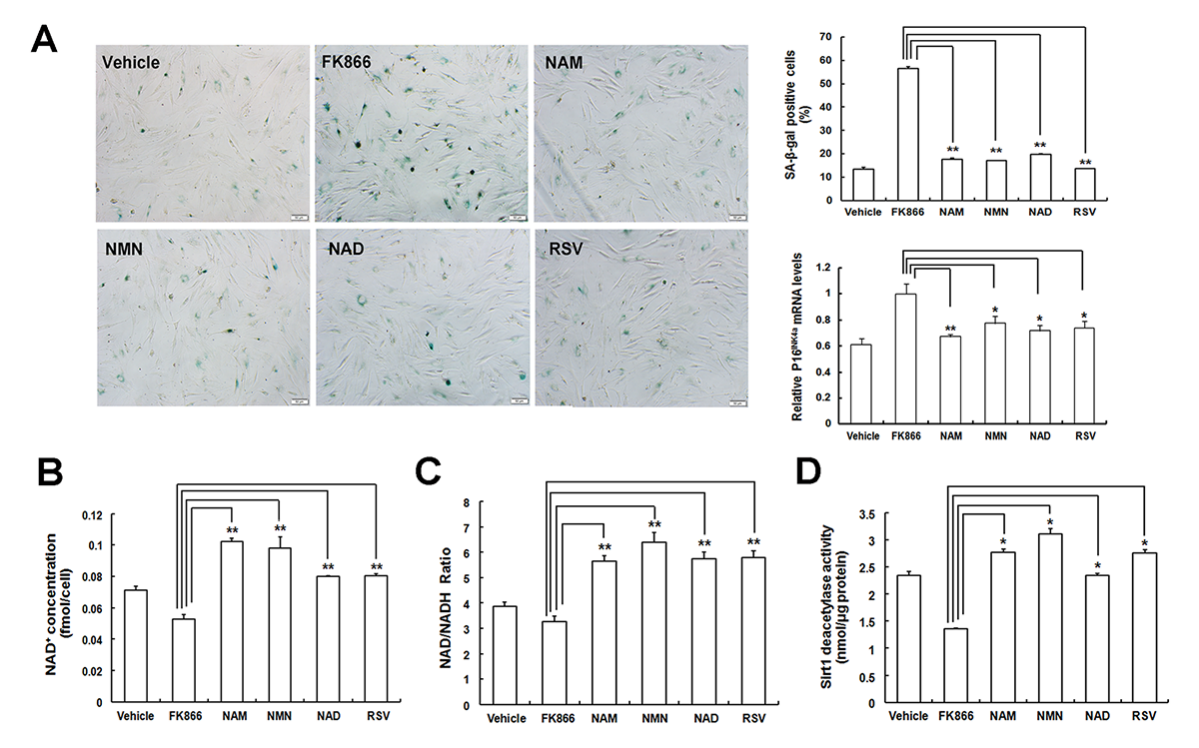
**NAD intermediates treatment attenuates FK866-induced MSC senescence. (A)** Evaluation of SA-β-gal staining and p16^INK4A^ expression in young EP MSCs with FK866 pre-treatment in the presence of Vehicle (DMSO treated), 100 μM NAM, 100 μM NMN, 100 μM NAD, and 5 μM RSV (scale bar = 50 μm) and the quantitative analysis of the percentages of SA-β-gal positive cells and p16^INK4A^ mRNA expression. **(B, C, D)** Effect of NAD intermediates treatment on NAD^+^ content **(B)** and NAD^+^/ NADH ratio **(C)**, as well as Sirt1 deacetylase activity **(D)** after pre-treating with FK866; n = 3 independent experiments. *P < 0.05, **P < 0.01.

Next, we also assessed the roles of different NAD intermediates in FK866-induced MSC senescence. SA-β-gal staining and quantitative analysis showed that the percentages of SA-β-gal-positive cells in young EP MSCs increased after treatment with FK866, and p16^INK4A^ mRNA expression also showed an upward trend in FK866-induced MSCs, whereas the addition of NAD intermediates, NAM, NMN, NAD, and RSV, significantly alleviated the augmented β-gal-positive cells and p16^INK4A^ expression induced by FK866 (Fig. 7A). Furthermore, NAD intermediates remarkably up-regulated FK866-induced reduction in the intracellular NAD^+^ content (Fig. 7B), as well as NAD^+^/NADH ratio (Fig. 7C), and Sirt1 activity (Fig. 7D). These data demonstrated that the supplementation of NAD intermediates could not only rescue replicative senescent MSCs but also ameliorate FK866-induced MSC senescence via preserving NAD^+^ levels and Sirt1 activity, further confirming that Nampt postpones MSC senescence via NAD^+^-Sirt1 mediation.

## DISCUSSION

Since Nampt was first reported to possess enzymatic activity in 1957 [28], its functions in individual aging, inflammation, cell metabolism, and other process have been intensively investigated [29–33]. In particular, there is abundant evidence that Nampt is closely related to cellular senescence in somatic cells. In mice, Nampt expression in the hippocampus decreased with aging, and treatment of FK866 lowered the intracellular NAD^+^ level in rat neuronal cells and induced cell death, which suggested that Nampt might regulate age-related brain diseases [34]. Van der Veer et al. [19] reported that Nampt over-expression attenuates replicative senescence and extends the life expectancy of human vascular smooth muscle cells, whereas FK866 induced premature senescence in these cells. Nonetheless, the relationship between Nampt and SC senescence is not well studied. The current study showed that Nampt mRNA and protein expression were significantly decreased in senescent LP MSCs compared to that in young EP MSCs, which is consistent with our previous data (not shown). In adult neural stem/progenitor cells, specific deletion of Nampt impairs cell proliferation and self-renewal during aging [35]. In addition, we previously showed that senescence in MSCs from aged rats contributes to the dramatic decrease in Nampt expression compared to that in young cells, during physiological senescence [22]. Thus, we speculated that Nampt might play a crucial regulatory role in MSC senescence.

Ample evidence suggests that senescent SCs have distinct features, including apparent morphological alterations, declined cell proliferation, and irreversible cell cycle arrest [36,37]. To test our hypothesis, we modulated Nampt expression through gene manipulation and pharmacological inhibition. Nampt depletion in young EP cells induced senescence-like alterations, including enlarged cell surface area and reduced cell aspect ratio. Cell proliferation significantly declined with the majority of cells arrested in G1 phase. These observations suggested that Nampt depletion gives rise to not only senescence-associated changes in morphology but also age-related alterations in biological characteristics. Another significant feature of adult SCs is their multi-lineage differentiation potential. Recently, Liu et al. reported that osteogenic and adipogenic differentiation of adipose-derived SCs gradually declined due to replicative senescence after serial passage *in vitro* [38]. Consistent herewith, our results showed that Nampt depletion inhibits osteogenesis and adipogenesis in young MSCs. Kim et al. found that continuous cultivation *in vitro* affects human MSC lineage fate determination by favoring adipogenesis at the expense of osteogenesis during aging, which partly contrasts our findings [39]. The discrepant findings might be attributed to model species variation and different cell culture and differentiation conditions. Studies confirming the effect of aging on lineage fate determination and comparing MSC adipogenesis and osteogenesis have not been performed to date, and further research is warranted.

Classic senescence-related SC markers, such as SA-β-gal activity and expression of the senescence-related factors p16^INK4A^ and p21^WAF1/CIP^, are commonly probed at cellular and mRNA levels to analyze cellular senescence [40,41]. To verify MSC replicative senescence at LP, we first carried out SA-β-gal staining. The SA-β-gal-positive cell fraction was significantly more abundant in Nampt-deficient MSCs than that in control cells, suggesting that Nampt suppression could induce or accelerate cellular senescence. Other biological indicators of cellular senescence include p16^INK4A^ and p21^WAF1/CIP^, which are cell-cycle related genes that participate in cell cycle modulation and function in a senescence-associated regulatory pathway [42–44]. Li et al. demonstrated that p21^WAF1/CIP^ and p16^INK4A^ expressions are elevated in aged muscle SCs compared to the levels in young cells [45]. In line herewith, we previously found that p16^INK4A^ and p21^WAF1/CIP^ levels were up-regulated in MSCs obtained from old rats compared to the levels in MSCs from young rats, with p16^INK4A^ expression showing the most potent response [22]. After serial passages, p16^INK4A^ mRNA expression was significantly up-regulated in senescent MSCs, although we did not find significant changes in p21^WAF1/CIP^ mRNA levels (data not shown). Moreover, accumulating studies have clarified that p16^INK4A^ serves as a reliable bio-marker to distinguish senescent cells, playing an important role in cellular senescence [46–49]. Therefore, in the current study, we examined SA-β-gal staining and p16^INK4A^ expression in detail as biological indicators to assess senescence. In line with the results of SA-β-gal staining and quantitative analyses, p16^INK4A^ expression was heightened in young MSCs after FK866 treatment or Nampt knockdown, which might account, at least in part, for MSC senescence induced by Nampt deprivation. We also analyzed Nampt-overexpressing LP MSCs. In contrast to depletion, Nampt repletion in senescent LP MSCs suppressed replicative senescence as evidenced by morphological, functional, and molecular changes, further strengthening our finding that Nampt exerts regulatory functions during the process of MSC replicative senescence.

The theory of aging in mammals proposed by Imai states that Nampt–NAD^+^–Sirt1 axis modulates aging, in which Nampt catalyzes the first step in transforming NAM and phosphoribosyl pyrophosphate into NMN [15]. Subsequently, NAD^+^ is synthesized by NMN adenylyltransferase (Namnt) using NMN and ATP [15,27,50]. Nampt indirectly regulates Sirt1 activity by influencing NAD^+^ biosynthesis, and subsequently, Sirt1 deacetylates many age-related signaling molecules [16,51,52]. Koltai et al. suggested that the age-related downregulation of intracellular NAD^+^ is associated with decreased Nampt expression [53]. Sirt1, a class III histone deacetylase, is one of the most important anti-aging proteins and the best-known member of the sirtuin family, which regulates cellular senescence [54,55]. In aged rodents, Sirt1 levels reportedly decrease significantly in the several tissues like liver, kidneys, heart, and lungs [56,57]. The enzymatic deacetylase activity of this NAD^+^-dependent longevity factor has been suggested to be modulated by the intracellular NAD^+^ content [51]. In our current study, both Sirt1 protein expression and Sirt1 activity in LP MSCs were substantially lower than those in EP MSCs. However, there was no significant change in Sirt1 protein  expression after FK866 treatment or Nampt over-expression in MSCs, whereas Nampt deficiency or adequate levels led to decreased or increased Sirt1 activity, respectively. The results were in line with our previous findings, indicating that the enzymatic activity is more important than the expression for Sirt1 functions [58–60]. Based on this notion, we hypothesized that Nampt could modulate intracellular NAD^+^ concentrations and Sirt1 activity in MSCs at EP or LP. As expected, NAD^+^ concentrations as well as NAD^+^/ NADH ratio were decreased, and Sirt1 activity was inhibited upon interference with Nampt expression in young MSCs. Accordingly, Nampt overexpression contributed to the accumulation of intracellular NAD^+^ level as well as NAD^+^/ NADH ratio, and also elevated Sirt1 activity in senescent MSCs. These results were in accordance with our previous findings in aged rats. NAM and NMN are important intermediates that are involved in enzymatic reactions during NAD^+^ biosynthesis. NAM, the amide form of vitamin B3, is a bi-functional molecular mediator that can either fuel NAD^+^ production or inhibit Sirt1 deacetylase activity; as such, it has contradictory roles in regulating cellular senescence. In particular, NAM can fuel NAD^+^ production at low doses and inhibit Sirt1 deacetylase at high doses [61]. In the current study, both replicative senescent MSCs and FK866-induced senescent cells were treated with different exogenous NAD intermediates, including NAM in the low concentration range (100 μM), 100 μM NMN, 100 μM NAD, and 5 μM of the Sirt1 activator RSV. Moreover, we verified that supplement of NAD intermediates could not only rescue replicative senescent MSCs but also ameliorate FK866-induced MSC senescence via preserving NAD^+^ levels and Sirt1 activity, further confirming that Nampt delays MSC senescence via the NAD^+^-Sirt1 axis. Our results were consistent with published data showing that NAM, an important NAD^+^ precursor, diminished FK866-induced cell senescence in human fibroblastic Hs68 cells [62]. The intermediate product, NMN, is the downstream effector of Nampt in the NAD^+^ salvage pathway, and exogenous NMN can increase intracellular NAD^+^ synthesis, which in turn can upregulate the diminished Sirt1 activity caused by aging [63]. However, in the current study, we used an exogenous NAD^+^ *in vitro* assay. Little is known about whether NAD^+^ from the cell lysate added to the reaction mixture is sufficient to increase the NAD^+^ concentration and alter Sirt1 activity. Therefore, the exact mechanism related to increased Sirt1 activity in an *in vitro* assay following NAD^+^ manipulation deserves deeper exploration in the future.

Overall, our data comprehensively validate the contention that Nampt has regulatory effects on MSC replicative senescence. Further, Nampt-mediated NAD synthesis and Sirt1 deacetylase activity are critical determinants of MSC senescence and the exogenous intermediates participated in NAD metabolism can delay or rescue Nampt-mediated MSC senescence. Our findings not only provide a foundation for further work to disclose the molecular mechanisms underlying SC senescence but might also aid in the development of promising strategies to delay MSC senescence and prevent age-related diseases. Further investigations concerning utilizing relevant animal models to understand the precise mechanisms involved in Nampt-mediated regulation on MSC senescence are in progress.

## MATERIALS AND METHODS

### Ethics Statement

This study was conducted in accordance with the ethical standards, the Declaration of Helsinki, and national and international guidelines, and was approved by the authors’ institutional review boards.

### MSC isolation and culture

MSCs from healthy, male, 1–2-month-old Wistar rats were isolated by the whole bone marrow adherent method, as previously described [22]. All animal experimental procedures used were consistent with the ethical standards of the Ethic Committee of Jilin University (permit number: SYXK 2013-0005). Cells were maintained in Dulbecco’s modified Eagle’s medium with nutrient mixture F-12 (DMEM-F12; Gibco, USA) with 10% fetal bovine serum (FBS, Gibco) 100 U/mL penicillin, and 100 μg/mL streptomycin. The culture medium was replaced every 3 days. MSCs were consecutively expanded up to P10. MSCs at P3 (EP) and P10 (LP) were used in subsequent experiments.

### Cell growth assays and population doubling time

The proliferative capacity of MSCs was assessed by the cell counting method. Briefly, 5 × 10^3^ cells were seeded into 24-well culture plates and were counted daily by trypan blue exclusion for 1 week. Cell growth curves were generated to analyze cell growth kinetics. PDT was calculated as previously described [22,23]. Briefly, 7 × 10^5^ cells were seeded in a 10-cm dish and cultured. When cells reached 80% confluency, they were harvested and counted. PDT was calculated according to the following formula: PDT = Ct / ln(Nf / Ni) / ln(2), where Nf is the number of harvested cells, Ni is the number of seeded cells, and Ct is the culture time.

### Cell cycle analysis

For cell cycle analysis, 1 × 10^6^ cells were harvested and fixed in 70% ethanol at 4 °C overnight. After centrifugation, the cell pellets were washed three times with ice-cold phosphate-buffered saline (PBS). Then, the pellets were incubated with 100 μL of RNaseA at 37 °C for 30 min. Next, the cells were incubated with 200 μL propidium iodide at 4 °C for at least 15 min. DNA content was analyzed using a FACS Calibur (BD Biosciences, USA) with Cell Quest software, and the SPF and PI were calculated.

### Adipogenic and osteogenic differentiation assays

To detect multilineage differentiation potential, MSCs were cultivated in adipogenic or osteogenic culture medium for 2–3 weeks, as previously described [23]. Oil red O staining was used to show lipid droplets after adipogenic induction, and Alizarin red S staining was conducted to observe bone matrix mineralization after osteogenic induction. To quantify the retention of Oil red O, stained adipocytes were extracted with 4% Igepal CA630 (Sigma-Aldrich, USA) in isopropanol for 15 min. Further, to quantify mineralization, 10% cetylpyridinium chloride (Sigma-Aldrich) was added for 30 min at room temperature (RT). Respective absorbance values were measured using a kinetics ELISA reader (Spectra MAX 250, Molecular Devices) at 490 nm and 560 nm for final quantitative analysis.

### Senescence-associated β-galactosidase activity assay

To assess MSC senescence, SA-β-gal staining was conducted using a senescent cell histochemical staining kit (Beyotime, China), following the manufacturer’s instruction. Briefly, cells were fixed in fixation buffer for 15 min at RT, washed twice with PBS, and incubated in Staining Solution Mix for 12–14 h. Then, the percentage of β-galactosidase-positive cells was determined using a bright-field microscope (OLYMPUS, Japan), assessing at least 200 cells in ten different microscopic fields.

### FK866 and NAD intermediates treatment

To determine the optimal concentration of FK866 without inducing robust cellular toxicity, 3,000 cells were seeded in 96-well plates and incubated in a humidified incubator at 37 °C for 24 h. Thereafter, the cells were treated with different concentrations of FK866 (0–100 nM; Sigma-Aldrich) for 72 h before the addition of CCK8 solution (10 μL/well). After incubation at 37 °C for 1 h, the absorbance at 450 nm was measured with a microplate reader. Subsequently, senescent LP MSCs were cultured in the presence or absence of different exogenous NAD intermediates, including 100 μM nicotinamide (NAM), 100 μM nicotinamide mononucleotide (NMN), 100 μM NAD, and 5 μM of Sirt1 activator resveratrol (RSV) for 48 h. For FK866-induced cellular senescence, young EP cells were pre-treated with complete medium containing 10 nM FK866 or Vehicle (DMSO) for 24 h, and then the cells following FK866 treatment were respectively exposed to 100 μM NAM, 100 μM NMN, 100 μM NAD, and 5 μM RSV for 48 h. Afterward, cells were collected for SA-β-gal assay, NAD^+^ and NAD^+^/NADH measurement, Sirt1 activity assay, and protein expression studies.

### Lentiviral transduction of MSCs

Prior to transduction, 1.5 × 10^4^ MSCs at P3 or P10 were seeded in 24-well plates and incubated at 37 °C overnight. Then, the cells were transduced with the purchased lentiviral particles encoding Rat Nampt or control vector, or with shNampt or non-targeting shCon (GeneChem, China) in the presence of 5 μg/mL polybrene (GeneChem) for 10 h. Seventy-two hours after transduction, EGFP expression was observed under a fluorescence microscope (OLYMPUS, Japan) and transduction efficiency was evaluated by RT-qPCR and western blotting.

### Gene expression analysis

Total RNA was extracted from MSCs using TRIzol (Takara, China). cDNA was synthesized using an RNA PCR Kit (AMV) Ver.3.0 (Takara). mRNA levels were measured by RT-qPCR using TransStart Top Green qPCR SuperMix (TRANS, China) with an ABI 7300 Real-Time PCR System (Applied Biosystems, USA). Rat-specific primers were synthesized, and included: β-actin: forward 5′-GGAGATTACTGCCCTGGCTCCTA-3′, reverse 5′-GACTCATCGTACTCCTGCTTGCTG-3′; Nampt: forward 5′-AGGGGCATCTGCTCATTTGG-3′, reverse 5′-TGGTACTGTGCTCTGCCGCT-3′; Sirt1: forward 5′-GCAGGTTGCAGGAATCCAAA-3′, reverse 5′-GGCAAGATGCTGTTGCAAAG-3′; p16^INK4A^: forward 5′-AAACACTTTCGGTCGTACCC-3′, reverse, 5′-GTCCTCGCAGTTCGAATC-3′. The thermal cycling protocol included pre-incubation at 95 °C for 2 min, followed by 40 cycles of amplification at 95 °C for 15 s and annealing for 30 s at 60 °C, and a final extension at 72 °C for 5 min. Data were normalized to the expression of β-actin using the 2^−ΔΔCt^ method.

### Western blot analysis

Total protein was extracted from MSCs using RIPA lysis buffer (Beyotime, China) supplemented with proteinase inhibitors. Protein concentrations were determined with a BCA Protein Assay Kit (Beyotime). Then, 25 μg of each protein sample was separated by 12% SDS-PAGE and transferred onto PVDF membranes (Millipore, USA) by electroblotting, after which nonspecific binding to the membrane was blocked with 5% non-fat milk for 1–2 h at RT. Then, the membranes were probed with anti-Sirt1 (1:1000, Upstate, USA), anti-Nampt (1:1000, BETHYL, USA), and anti-β-actin (1:1500, Abcam, UK) antibodies diluted in Tris-buffered saline. After incubation at 4 °C overnight, the membranes were incubated with anti-rabbit IgG secondary antibody. The protein blots were visualized using an Electro-Chemi-Luminescence detection system (JENE, UK) and quantified with ImageJ software.

### Quantification of intracellular NAD^+^ and NAD^+^/NADH ratio

Intracellular NAD^+^ concentrations were determined using the NAD^+^/NADH Quantification Colorimetric Kit (BioVision, USA), in accordance with the manufacturer’s instructions. Briefly, 400 μL NADH/ NAD^+^ Extraction Buffer was added to 2 × 10^5^ cells frozen at −80 °C for 20 min and thawed at RT for 10 min. Then, half of the supernatants were directly transferred to a 96-well plate, and the other half were heated in a water bath at 60 °C for 30 min before the detection of NADt (NADH and NAD^+^) and NADH, respectively. Next, 98 μL of NAD^+^ Cycling Buffer and 2 μL of NAD^+^ Cycling Enzyme Mix were gently mixed and added to each well for incubation at 37 °C for 5 min. Then, 10 μL of NADH Developer was added for 2 h, and the optical density at 450 nm was read using a multi-well spectrophotometer. The NAD^+^ and NADH amount, and NAD^+^/NADH ratio in each sample were calculated and normalized by cell numbers. A standard curve was performed with the NADH standards included in the kit.

### Sirt1 deacetylase activity assay

Sirt1 deacetylase activity was measured using a SIRT1 assay kit (Sigma-Aldrich) following the manufacturer’s instructions. Total protein was extracted, and protein concentrations were measured as described above. Next, 20 μL of protein extract was gently and uniformly blended with a mixture of 15 μL Assay Buffer and 5 μL NAD^+^ solution. Then, 10 μL SIRT1 Substrate Solution was added to the mixture, which was incubated at RT for 10 min. After the addition of 5 μL of developing solution, the samples were incubated at 37 °C for 10 min. Fluorescence was read at 450 nm (excitation 360 nm) using a plate reader, and Sirt1 activity was calculated and normalized by protein content. A standard curve was generated on the basis of the Standard Solution contained in the kit.

### Statistical analysis

All experiments were performed three times independently. Two groups were compared by a two-tailed Student’s t-test. All experimental data are expressed as the mean ± standard deviation, and differences were considered significant at *P* < 0.05.

## SUPPLEMENTARY MATERIAL

Supplementary Figure
