# Supplementary material for: Nicotinamide phosphoribosyltransferase postpones rat bone marrow mesenchymal stem cell senescence by mediating NAD+–Sirt1 signaling
Source: Aging (Albany NY). 2019 Jun 7;11(11):3505–22. doi: 10.18632/aging.101993 (PMC6594813; doi:10.18632/aging.101993)
Supplement: Supplementary Figure [file aging-11-101993-s001.pdf]

SUPPLEMENTARY MATERIAL

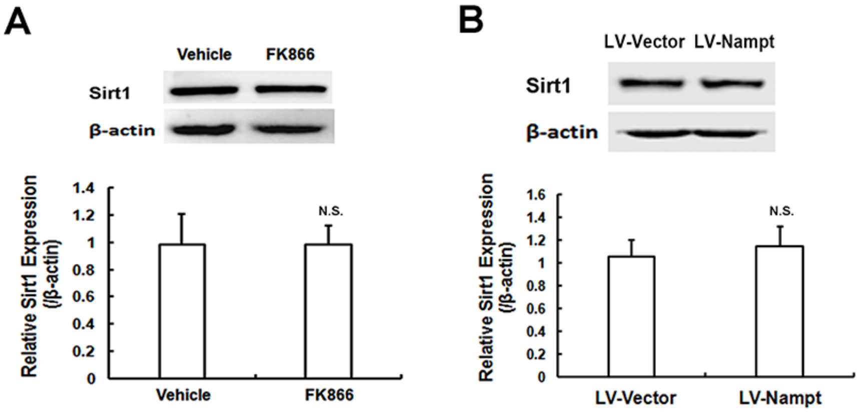

Supplementary Figure 1. Determination of Sirt1 protein expression by Western blotting. (A) The effect of the specific Nampt inhibitor FK866 on Sirt1 protein expression in young EP MSCs. **(B)** The protein levels of Sirt1 in senescent LP MSCs when Nampt was over-expressed; n = 3 independent experiments. N.S., not significant.
